# Voice-based assessments of trustworthiness, competence, and warmth in blind and sighted adults

**DOI:** 10.3758/s13423-016-1146-y

**Published:** 2016-10-13

**Authors:** Anna Oleszkiewicz, Katarzyna Pisanski, Kinga Lachowicz-Tabaczek, Agnieszka Sorokowska

**Affiliations:** 1 Institute of Psychology, University of Wroclaw, Wroclaw, Poland; 2 Smell & Taste Clinic, Department of Otorhinolaryngology, TU Dresden, Dresden, Germany; 3 School of Psychology, University of Sussex, Brighton, United Kingdom

**Keywords:** Voice perception, Social perception, Sightedness, Blind, Nonverbal communication

## Abstract

The study of voice perception in congenitally blind individuals allows researchers rare insight into how a lifetime of visual deprivation affects the development of voice perception. Previous studies have suggested that blind adults outperform their sighted counterparts in low-level auditory tasks testing spatial localization and pitch discrimination, as well as in verbal speech processing; however, blind persons generally show no advantage in nonverbal voice recognition or discrimination tasks. The present study is the first to examine whether visual experience influences the development of social stereotypes that are formed on the basis of nonverbal vocal characteristics (i.e., voice pitch). Groups of 27 congenitally or early-blind adults and 23 sighted controls assessed the trustworthiness, competence, and warmth of men and women speaking a series of vowels, whose voice pitches had been experimentally raised or lowered. Blind and sighted listeners judged both men’s and women’s voices with lowered pitch as being more competent and trustworthy than voices with raised pitch. In contrast, raised-pitch voices were judged as being warmer than were lowered-pitch voices, but only for women’s voices. Crucially, blind and sighted persons did not differ in their voice-based assessments of competence or warmth, or in their certainty of these assessments, whereas the association between low pitch and trustworthiness in women’s voices was weaker among blind than sighted participants. This latter result suggests that blind persons may rely less heavily on nonverbal cues to trustworthiness compared to sighted persons. Ultimately, our findings suggest that robust perceptual associations that systematically link voice pitch to the social and personal dimensions of a speaker can develop without visual input.

## Acoustic perception in blind persons

Three key hypotheses compete with each other regarding general acoustic perception in blind persons (Kupers & Ptito, 2014). The first of these states that blind persons may possess degraded acoustic perception relative to sighted persons, particularly if visual experience is necessary to calibrate the other senses. This first hypothesis has not garnered a great deal of support, as it is now known that congenital blindness or the loss of vision early in life can cause substantial structural reorganization of the brain, wherein the structures typically specialized for vision are recruited for the processing of stimuli in other modalities, including audition, allowing normal hearing to develop without vision (reviewed in Kupers & Ptito, 2014; Rauschecker, 1995). Hence, a second hypothesis posits that blind persons process sounds similarly to sighted persons, although this ability may develop through alternative mechanisms. A third, and the most recent, hypothesis posits that blind persons may possess “supra-normal” nonvisual sensory capabilities, as a result of either perceptual learning (Gagnon, Ismaili, Ptito, & Kupers, 2015) or the reorganization of various brain areas (e.g., the occipital cortex; Leclerc, Saint-Amour, Lavoie, Lassonde, & Lepore, 2000), suggesting that blind persons may outperform their sighted counterparts in nonvisual auditory tasks. The latter hypothesis has gained support from studies testing spatial sound localization (Fieger, Röder, Teder-Sälejärvi, Hillyard, & Neville, 2006), simple tone discrimination (Gougoux et al., 2004), and human echolocation (Schenkman & Nilsson, 2010).

## The special case of voice perception

Previous findings on general acoustic perception in blind persons cannot, however, be directly applied to voice perception. Indeed, compared to nonvocal sounds, vocalizations are acoustically complex, broadband, and typically periodic signals (Titze, 1994), and are selectively processed in higher-level regions of the auditory cortex near the superior temporal sulcus (Belin, Fecteau, & Bédard, 2004; Belin, Zatorre, & Ahad, 2002; Pernet et al., 2015). Perhaps most crucially, voice perception often involves complex social cognition. Voices play a critical role in everyday nonverbal communication, allowing us not only to readily recognize familiar others, but also to gauge a speaker’s sex, age, body size, ethnicity, social status, and emotional or motivational state (Kreiman & Sidtis, 2011; Pisanski et al., 2014; Puts, Jones, & DeBruine, 2012).

Even within the category of vocal sounds, listeners process voices differently when attending to verbal (i.e., speech) versus nonverbal (i.e., social and indexical) information from the voice. The neural processing of verbal and nonverbal vocal sounds appears to involve functionally divergent pathways in the brain (Belin, Bestelmeyer, Latinus, & Watson, 2011). Importantly, although behavioral studies have provided some evidence for superior verbal or speech processing abilities in blind persons—for example, in verbal memory (Amedi, Raz, Pianka, Malach, & Zohary, 2003) and speech sound discrimination (Hugdahl et al., 2004; Muchnik, Efrati, Nemeth, Malin, & Hildesheimer, 1991) tasks—blind persons generally show no advantage in nonverbal voice recognition or discrimination tasks (Gougoux et al., 2009; Günzburger, Bresser, & Keurs, 1987; Winograd, Kerr, & Spence, 1984; but see Bull, Rathborn, & Clifford, 1983). Moreover, blind persons perform no better or worse than their sighted counterparts in estimating the relative heights of men using only nonverbal voice cues (Pisanski, Oleszkiewicz, & Sorokowska, 2016). Thus, although there is some evidence that blind persons may process voices differently from sighted persons, these differences appear to arise predominantly in the processing of verbal rather than nonverbal information.

## Voice-based assessments of social traits

To our knowledge, the present study is the first to examine whether blind and sighted persons differ in their judgments of social character traits based solely on nonverbal voice cues. The most salient nonverbal feature of the human voice is pitch, the perceptual correlate of fundamental frequency (*F*0) and its harmonics (Titze, 1994). Voice pitch is determined by the rate of vocal-fold vibration, which in turn is influenced by pubertal and circulating levels of testosterone that affect the length of the vocal folds (Dabbs & Mallinger, 1999; Harries, Walker, Williams, Hawkins, & Hughes, 1997). Voice pitch is highly sexually dimorphic and changes systematically throughout the lifetime, thereby reliably signaling an individual’s sex and general age (Kreiman & Sidtis, 2011).

Voice pitch also influences listeners’ assessments of various socially relevant traits. Recent studies have focused on traits that are particularly important in a mating context, such as attractiveness, masculinity or femininity, and dominance (reviewed in Kreiman & Sidtis, 2011; Pisanski & Bryant, 2016; Puts et al., 2012). However, within a broader social context, competence and warmth are considered universal dimensions of social perception (see Fiske, Cuddy, & Glick, 2007, for a review). These dimensions explain more than 80 % of the variance in our personality judgments of others (Wojciszke, Bazinska, & Jaworski, 1998). *Competence* broadly reflects traits related to ability, such as dominance and intelligence, whereas *warmth* reflects perceived intent, including sincerity and kindness.

In addition to competence and warmth, we also investigated the effect of voice pitch on perceived *trustworthiness*. Trustworthiness may be particularly important for blind persons, who must routinely rely on the opinions and assistance of others in everyday life (Lewis & Weigert, 1985), but who cannot rely on visual (e.g., facial; Oosterhof & Todorov, 2008) indicators of trustworthiness. Moreover, although trustworthiness has often been discussed as an element of warmth (Fiske et al., 2007), it remains unknown whether voice pitch affects judgments of warmth and trustworthiness in the same way. Indeed, studies with sighted listeners have indicated that speakers with low voice pitch are typically perceived as being more competent and trustworthy than speakers with higher voice pitch (Klofstad, Anderson, & Nowicki, 2015; McAleer, Todorov, & Belin, 2014; Tigue, Borak, O’Connor, Schandl, & Feinberg, 2012; Tsantani, Belin, Paterson, & McAleer, 2016). In contrast, studies examining voice-based assessments of warmth have produced equivocal results (Berry, 1991; Hughes, Pastizzo, & Gallup, 2008; Ko, Judd, & Stapel, 2009; McAleer et al., 2014). Recently, McAleer et al. reported a positive relationship between listeners’ voice-based judgments of warmth and trustworthiness, both of which correlated negatively with judgments of competence and dominance. However, like previous studies, the researchers did not experimentally manipulate voice pitch.

In the present study, we predicted that blind listeners would associate low voice pitch with relatively higher competence and trustworthiness, and that the strength of this association would be similar among blind and sighted listeners. We made no a priori predictions regarding assessments of warmth, for which the results among sighted listeners in previous studies have been mixed.

## Method

### Voice stimuli

Voice recordings were conducted in a sound-controlled booth using a Sennheiser condenser microphone with a cardioid pick-up pattern and at a distance of 5–10 cm. Recordings were obtained from four men and four women speaking the monophthong vowels /ɑ/, /i/, /ɛ/, /o/, and /u/. The audio was digitally encoded at a sampling rate of 96 kHz and 32-bit amplitude quantization and was stored on a computer as WAV files. The voice editing and manipulation was performed in Praat, version 5.2.15 (Boersma & Weenink, 2015). Vowels were first separated by 200 ms of silence. The pitch of each voice was than raised or lowered by adding or subtracting 0.75 equivalent rectangular bandwidths (ERBs) of the baseline *F*0, creating high- and low-pitch versions of each voice (Table 1). The ERB scale is pseudologarithmic and controls for the difference between *F*0 and perceived pitch. All voice stimuli were amplitude-normalized to 70 dB RMS SPL.

**Table 1:** Voice pitch (measured as fundamental frequency, *F*0) of voice stimuli following manipulation

	Pitch Manipulation	Pitch (*F*0, Hz)
Female voices	Raised	251.1
	Lowered	190.2
Male voices	Raised	137.3
	Lowered	81.5

### Participants

Fifty men and women participated in the study, including 27 healthy blind adults (17 females, 10 males; 24–65 years of age, *M* = 37.9 ± 11.1 years) and 23 age-matched controls with normal vision (15 females, 8 males; 20–65 years of age, *M* = 38.7 ± 14.5 years). All but two of the blind participants had been blind since birth (congenitally blind), whereas two women had lost their sight in the first month of life (early blind; Rombaux et al., 2010). All participants reported normal hearing and no neurological impairments, provided written informed consent, and were compensated for their participation. The study was performed in accordance with the Declaration of Helsinki on Biomedical Studies Involving Human Subjects and was approved by the University of Wroclaw Institutional Review Board.

### Procedure

Participants completed the experiment in individual sessions. A standardized interview was first used to collect demographic information and to confirm the absence of hearing disorders, head injuries or diseases, and the use of medication that could influence hearing. Participants were then randomly assigned to assess either male or female voices. They were instructed that they would hear a series of voices, and that after each voice they would be asked to assess the person speaking on one of three traits (competence, trustworthiness, or warmth) using a 7-point scale, ranging from 1 (*s/he definitely is not*) to 7 (*s/he definitely is*)*.* Participants assessed each voice stimulus on each of the three traits; trials were blocked by trait, and the presentation order of blocks, as well as of the voice stimuli within each block, was fully randomized. Voices were presented to participants via a custom computer interface and through Sennheiser HD-280 PRO headphones. The experimenter executed the experiment and inputted participants’ verbal responses, which automatically loaded the next trial. Each participant rated 24 voices in total, and the entire task took approximately 10 min.

To create identical testing conditions, sighted participants were asked to close their eyes during the experiment, and all participants were seated with their backs to the computer. Following the auditory task, all participants were asked to rate the extent to which they were confident in their judgments on a scale from 1 (*not at all*) to 7 (*completely*)*.*

## Results

We conducted a series of linear mixed models (LMMs) with maximum-likelihood estimation, one for each sex of the voice and for each trait. Listener ID was included as a random subject variable, Sex of Listener as a random factor, and Sightedness (blind, sighted) and Voice Pitch Manipulation (raised, lowered) as fixed factors. A Mann–Whitney *U* test revealed no difference in confidence judgments between sighted and blind participants (*U* = 234.5, *p* = .13).

### Men’s voices

The LMM examining listeners’ assessments of men’s competence revealed a main effect of voice pitch manipulation [*F*(1, 146) = 6.03, *p* = .015], wherein lowered-pitch voices were rated as being more competent than raised-pitch voices (Table 2). We found no other main or interaction effects (all *F*s < 0.66, all *p*s > .42). The model examining assessments of men’s warmth revealed no main or interaction effects (all *F*s < 0.3, all *p*s > .58). Finally, the model examining assessments of trustworthiness revealed a main effect of voice pitch manipulation [*F*(1, 146) = 8.8, *p* = .004], wherein lowered-pitch voices were rated as being more trustworthy than raised-pitch voices, with no other main or interaction effects (all *F*s < 1.15, all *p*s > .28).

**Table 2:** Voice-based assessments of competence, warmth, and trustworthiness by sighted and blind adults

	Raised Pitch				Lowered Pitch			
	Female Voices		Male Voices		Female Voices		Male Voices	
	Sighted	Blind	Sighted	Blind	Sighted	Blind	Sighted	Blind
Competence								
*M*	3.50	3.42	3.38	3.70	4.75	4.20	3.95	4.27
*SD*	1.085	1.362	1.237	1.042	0.890	1.154	0.941	0.932
*n*	13	15	10	11	13	15	10	11
Warmth								
*M*	4.58	4.18	4.05	4.07	4.21	3.70	3.88	4.02
*SD*	0.632	1.144	1.039	1.084	1.015	1.158	1.120	1.252
*n*	13	15	10	11	13	15	10	11
Trustworthiness								
*M*	3.77	3.65	3.38	3.73	4.83	3.95	4.20	4.11
*SD*	1.214	0.949	0.680	1.339	0.753	0.769	0.864	1.348
*n*	13	15	10	11	13	15	10	11

*M* = mean, *SD* = standard deviation, *n* = sample size

### Women’s voices

The LMM examining listeners’ assessments of women’s competence revealed a main effect of voice pitch manipulation [*F*(1, 201) = 30.2, *p* < .001], wherein lowered-pitch voices were rated as being more competent than raised-pitch voices. No other main or interaction effects emerged (all *F*s < 0.66, all *p*s > .42). The model examining assessments of warmth revealed a main effect of voice pitch manipulation [*F*(1, 201) = 8.6, *p* = .004], wherein raised-pitch voices were rated as being warmer than lowered-pitch voices, with no other effects (all *F*s < 2.0, all *p*s > .17). Finally, the model examining assessments of trustworthiness revealed main effects of both voice pitch manipulation [*F*(1, 201) = 9.5, *p* = .002] and sightedness [*F*(1, 27) = 4.9, *p* = .035], as well as an interaction between pitch manipulation and sightedness [*F*(1, 201) = 4.6, *p* = .033]. Here, lowered-pitch voices were assessed as being more trustworthy than raised-pitch voices, and this effect of pitch on assessments of women’s trustworthiness was greater for sighted than for blind participants (Table 2).

## Discussion

Social judgments represent an adaptation to life in a group. Effectively assessing the unobservable character traits of others on the basis of limited information helps us determine whether someone is a friend or foe (Fiske et al., 2007) or a suitable potential partner (Puts et al., 2012). Until now, this ability has only been investigated in sighted persons. Our results indicate that both blind and sighted adults judged men’s and women’s voices with experimentally lowered pitch as significantly more competent and more trustworthy than those same voices with raised pitch. In contrast, raised voice pitch was associated with warmth, but only for women’s voices. Critically, manipulations of voice pitch elicited analogous assessments of competence and warmth, regardless of whether the listener was sighted or blind. Differences between blind and sighted persons emerged only in judgments of women’s trustworthiness, wherein the association between low pitch and trustworthiness was stronger among sighted than among blind participants.

Studies have generally failed to show differences between sighted and blind adults in voice recognition tasks (Gougoux et al., 2009; Günzburger et al., 1987; Winograd et al., 1984). Blind persons can also estimate body size from the voice as accurately as can sighted persons (Pisanski et al., 2016). In line with these previous findings, our study provides novel evidence that blind persons process socially relevant information (i.e., competence and warmth) from nonverbal voice cues similarly to sighted persons. There are several potential and non-mutually-exclusive explanations for this finding. At a fundamental level, assessing others on the basis of their vocalizations represents an evolutionarily primitive and ecologically relevant ability that is widespread among vocalizing mammals (Taylor & Reby, 2010), and therefore may be innate or may require little or no visual experience to develop. Both sighted and blind persons may, for instance, be capable of gathering reliable social information from a person’s behavioral patterns, and consequently may learn to form general associations between specific social traits and vocal traits even in the absence of visual information. The associations between voice pitch and social traits may also stem from general perceptual biases. Indeed, cross-modal pitch correspondences have been observed across human cultures and are highly general, applying to tonal, musical, and vocal pitch (Eitan & Timmers, 2010; Ohala, 1984; Parise, 2016).

Voice pitch did, however, have a stronger effect on sighted than on blind listeners’ assessments of women’s trustworthiness. This finding is of interest for two reasons. First, it suggests that blind persons may rely less heavily on nonverbal cues to trustworthiness compared to sighted persons, potentially because blind persons might be more cautious when deciding whether to rely on someone. Second, this finding suggests that voice-based assessments of trustworthiness and warmth, although closely related (McAleer et al., 2014), may enjoy some degree of independence.

Women’s voices with raised pitch were judged as being relatively warmer than voices with lowered pitch, whereas voice pitch did not influence judgments of men’s warmth. Ko et al. (2009) similarly reported that although job applicants with masculine voices were judged as being more competent, vocal masculinity/femininity did not predict judgments of warmth. Warmth entails “other-profitable” traits with immediate benefits to the assessor, as well as costs if it is misjudged (Fiske et al., 2007). Thus, people may require cues from multiple modalities to judge the warmth of others, particularly of men. However, evidence that warmth-related judgments are formed on minimal premises and significantly faster than competence-related judgments suggests that this is unlikely (Ybarra, Chan, & Park, 2001). Alternatively, high voice pitch may activate connotations with femininity that are related to perceived warmth (see, e.g., Eagly & Mladinic, 1989) and that may have conflicting and cancelling effects on warmth judgments of men. Additional research will be needed to disambiguate the effect of voice pitch on warmth judgments and its potential interaction with the sex of the speaker.

In the present study, we manipulated the pitch of voices speaking vowel sounds, further indicating that a single and briefly presented nonverbal vocal parameter can provide a clear premise for social inferences (McAleer et al., 2014). Listeners judged each voice independently using a Likert scale (following, e.g., Feinberg, Jones, Little, Burt, & Perrett, 2005; Pisanski & Rendall, 2011). Although some previous studies have used a two-alternative forced-choice rating paradigm (e.g., Jones, Feinberg, DeBruine, Little, & Vukovic, 2010; Tsantani et al., 2016) or a scale/forced-choice hybrid (e.g., Vukovic et al., 2011), a Likert scale allows for more variance in listeners’ responses than does a binary task, and therefore may increase the likelihood of uncovering potentially subtle group differences.

Our findings suggest that blind persons assess character traits on the basis of a person’s voice pitch in a similar way as do sighted persons, and that such associations can therefore develop without visual input. Thus, although the present study extends our limited knowledge about social perception in blind persons, it also offers novel insight into the potential mechanisms and development of social voice perception more generally. Given that voice pitch influences critical social decisions such as which leaders we choose to vote for (Klofstad et al., 2015; Tigue et al., 2012) or which candidates we hire following a job interview (Schroeder & Epley, 2015), understanding how these vocal stereotypes develop is paramount.

## References

[CR1] Amedi A, Raz N, Pianka P, Malach R, Zohary E (2003). Early “visual” cortex activation correlates with superior verbal memory performance in the blind. Nature Neuroscience.

[CR2] Belin P, Bestelmeyer PEG, Latinus M, Watson R (2011). Understanding voice perception. British Journal of Psychology.

[CR3] Belin P, Fecteau S, Bédard C (2004). Thinking the voice: Neural correlates of voice perception. Trends in Cognitive Sciences.

[CR4] Belin P, Zatorre RJ, Ahad P (2002). Human temporal-lobe response to vocal sounds. Cognitive Brain Research.

[CR5] Berry DS (1991). Accuracy in social perception: Contributions of facial and vocal information. Journal of Personality and Social Psychology.

[CR6] Boersma, P., & Weenink, D. (2015). Praat: Doing phonetics by computer (Version 5.2.15) [Computer program]. Retrieved from www.praat.org

[CR7] Bull R, Rathborn H, Clifford BR (1983). The voice-recognition accuracy of blind listeners. Perception.

[CR8] Dabbs JM, Mallinger A (1999). High testosterone levels predict low voice pitch among men. Personality and Individual Differences.

[CR9] Eagly AH, Mladinic A (1989). Gender stereotypes and attitudes toward women and men. Personality and Social Psychology Bulletin.

[CR10] Eitan Z, Timmers R (2010). Beethoven’s last piano sonata and those who follow crocodiles: Cross-domain mappings of auditory pitch in a musical context. Cognition.

[CR11] Feinberg DR, Jones BC, Little AC, Burt DM, Perrett DI (2005). Manipulations of fundamental and formant frequencies influence the attractiveness of human male voices. Animal Behaviour.

[CR12] Fieger A, Röder B, Teder-Sälejärvi W, Hillyard SA, Neville HJ (2006). Auditory spatial tuning in late-onset blindness in humans. Journal of Cognitive Neuroscience.

[CR13] Fiske ST, Cuddy AJC, Glick P (2007). Universal dimensions of social cognition: Warmth and competence. Trends in Cognitive Sciences.

[CR14] Gagnon L, Ismaili ARA, Ptito M, Kupers R (2015). Superior orthonasal but not retronasal olfactory skills in congenital blindness. PLoS ONE.

[CR15] Gougoux F, Belin P, Voss P, Lepore F, Lassonde M, Zatorre RJ (2009). Voice perception in blind persons: A functional magnetic resonance imaging study. Neuropsychologia.

[CR16] Gougoux F, Lepore F, Lassonde M, Voss P, Zatorre RJ, Belin P (2004). Neuropsychology: Pitch discrimination in the early blind. Nature.

[CR17] Günzburger D, Bresser A, Keurs MT (1987). Voice identification of prepubertal boys and girls by normally sighted and visually handicapped subjects. Language and Speech.

[CR18] Harries MLL, Walker JM, Williams DM, Hawkins S, Hughes IA (1997). Changes in the male voice at puberty. Archives of Disease in Childhood.

[CR19] Hugdahl K, Ek M, Takio F, Rintee T, Tuomainen J, Haarala C, Hämäläinen H (2004). Blind individuals show enhanced perceptual and attentional sensitivity for identification of speech sounds. Cognitive Brain Research.

[CR20] Hughes SM, Pastizzo MJ, Gallup GG (2008). The sound of symmetry revisited: Subjective and objective analyses of voice. Journal of Nonverbal Behavior.

[CR21] Jones BC, Feinberg DR, DeBruine LM, Little AC, Vukovic J (2010). A domain-specific opposite-sex bias in human preferences for manipulated voice pitch. Animal Behaviour.

[CR22] Klofstad CA, Anderson RC, Nowicki S (2015). Perceptions of competence, strength, and age influence voters to select leaders with lower-pitched voices. PLoS ONE.

[CR23] Ko SJ, Judd CM, Stapel DA (2009). Stereotyping based on voice in the presence of individuating information: Vocal femininity affects perceived competence but not warmth. Personality and Social Psychology Bulletin.

[CR24] Kreiman, J., & Sidtis, D. (2011). *Foundations of voice studies: An interdisciplinary approach to voice production and perception*. Hoboken: Wiley-Blackwell. Retrieved from http://eu.wiley.com/WileyCDA/WileyTitle/productCd-0631222979.html

[CR25] Kupers R, Ptito M (2014). Compensatory plasticity and cross-modal reorganization following early visual deprivation. Neuroscience & Biobehavioral Reviews.

[CR26] Leclerc C, Saint-Amour D, Lavoie ME, Lassonde M, Lepore F (2000). Brain functional reorganization in early blind humans revealed by auditory event‐related potentials. NeuroReport.

[CR27] Lewis JD, Weigert A (1985). Trust as a social reality. Social Forces.

[CR28] McAleer P, Todorov A, Belin P (2014). How do you say “hello”? Personality impressions from brief novel voices. PLoS ONE.

[CR29] Muchnik C, Efrati M, Nemeth E, Malin M, Hildesheimer M (1991). Central auditory skills in blind and sighted subjects. Scandinavian Audiology.

[CR30] Ohala JJ (1984). An ethological perspective on common cross-language utilization of F0 of voice. Phonetica.

[CR31] Oosterhof NN, Todorov A (2008). The functional basis of face evaluation. Proceedings of the National Academy of Sciences.

[CR32] Parise CV (2016). Crossmodal correspondences: Standing issues and experimental guidelines. Multisensory Research.

[CR33] Pernet CR, McAleer P, Latinus M, Gorgolewski KJ, Charest I, Bestelmeyer PE, Belin P (2015). The human voice areas: Spatial organization and inter-individual variability in temporal and extra-temporal cortices. NeuroImage.

[CR34] Pisanski, K., & Bryant, G. A. (2016). The evolution of voice perception. In N. S. Eidsheim & K. L. Meizel (Eds.), *The Oxford handbook of voice studies*. New York: Oxford University Press (in press).

[CR35] Pisanski K, Fraccaro PJ, Tigue CC, O’Connor JJM, Röder S, Andrews PW, Feinberg DR (2014). Vocal indicators of body size in men and women: A meta-analysis. Animal Behaviour.

[CR36] Pisanski K, Oleszkiewicz A, Sorokowska A (2016). Can blind persons accurately assess body size from the voice?. Biology Letters.

[CR37] Pisanski K, Rendall D (2011). The prioritization of voice fundamental frequency or formants in listeners’ assessments of speaker size, masculinity, and attractiveness. Journal of the Acoustical Society of America.

[CR38] Puts D, Jones BC, DeBruine LM (2012). Sexual selection on human faces and voices. Journal of Sex Research.

[CR39] Rauschecker JP (1995). Compensatory plasticity and sensory substitution in the cerebral cortex. Trends in Neurosciences.

[CR40] Rombaux P, Huart C, De Volder AG, Cuevas I, Renier L, Duprez T, Grandin C (2010). Increased olfactory bulb volume and olfactory function in early blind subjects. NeuroReport.

[CR41] Schenkman BN, Nilsson ME (2010). Human echolocation: Blind and sighted persons’ ability to detect sounds recorded in the presence of a reflecting object. Perception.

[CR42] Schroeder J, Epley N (2015). The sound of intellect: Speech reveals a thoughtful mind, increasing a job candidate’s appeal. Psychological Science.

[CR43] Taylor AM, Reby D (2010). The contribution of source-filter theory to mammal vocal communication research: Advances in vocal communication research. Journal of Zoology.

[CR44] Tigue CC, Borak DJ, O’Connor JJM, Schandl C, Feinberg DR (2012). Voice pitch influences voting behavior. Evolution and Human Behavior.

[CR45] Titze IR (1994). Principles of vocal production.

[CR46] Tsantani MS, Belin P, Paterson HM, McAleer P (2016). Low vocal pitch preference drives first impressions irrespective of context in male voices but not in female voices. Perception.

[CR47] Vukovic J, Jones BC, Feinberg DR, DeBruine LM, Smith FG, Welling LLM, Little AC (2011). Variation in perceptions of physical dominance and trustworthiness predicts individual differences in the effect of relationship context on women’s preferences for masculine pitch in men’s voices: Vocal attractiveness, trust and dominance. British Journal of Psychology.

[CR48] Winograd E, Kerr NH, Spence MJ (1984). Voice recognition: Effects of orienting task, and a test of blind versus sighted listeners. American Journal of Psychology.

[CR49] Wojciszke B, Bazinska R, Jaworski M (1998). On the dominance of moral categories in impression formation. Personality and Social Psychology Bulletin.

[CR50] Ybarra O, Chan E, Park D (2001). Young and old adults’ concerns about morality and competence. Motivation and Emotion.

